# DRESS syndrome: an interaction between drugs, latent viruses, and the immune system^☆^

**DOI:** 10.1016/j.abd.2023.12.010

**Published:** 2024-11-08

**Authors:** Paulo Ricardo Criado, Mayra Ianhez, Hélio Amante Miot, Roberta Fachini Jardim Criado, Carolina Talhari, Paulo Müller Ramos

**Affiliations:** a Centro Universitário Faculdade de Medicina do ABC, Santo André, SP, Brazil; b Faculdade de Ciências Médicas de Santos, (Fundação Lusíada), Santos, SP, Brazil; c Department of Dermatology, Hospital de Doenças Tropicais de Goiás, Goiânia, GO, Brazil; d Department of Infectology, Dermatology, Imaging Diagnosis and Radiotherapy, Faculty of Medicine, Universidade Estadual Paulista, Botucatu, SP, Brazil; e Alergoskin Alergia e Dermatologia, UCARE Center and ADCARE, Santo André, SP, Brazil; f Department of Dermatology, Universidade do Estado do Amazonas, Manaus, AM, Brazil

**Keywords:** Drug hypersensitivity syndrome, Drug eruption, Drug-related side effects and adverse reactions, Eosinophilia, Substance and drug-induced liver disease

## Abstract

Drug-induced hypersensitivity syndrome, also known as DRESS syndrome, is a serious and potentially fatal reaction that occurs in response to prolonged use (generally between 14 and 60 days) of certain drugs, and which has no predilection for gender or age group. It is believed that DRESS syndrome has a genetic basis and results from the interaction between metabolites of certain pharmacological groups, reactivation of latent viruses (especially from the *Herpesviridae* family), and a cellular immune response. The classic manifestation of DRESS syndrome includes a generalized rash accompanied by fever, eosinophilia, lymphadenopathy, and systemic involvement such as hepatitis, nephritis, or pneumonitis. With the continuous increase in the availability of drugs and the aging of the population, there is a favorable scenario for the development of adverse drug reactions. Physicians should be prepared for the early diagnosis of DRESS syndrome, the identification and immediate suspension of the drug involved, and also manage systemic involvement, which may require prolonged immunosuppressive therapy. This article provides an update on the clinical, physiopathological and therapeutic aspects of DRESS syndrome.

## Introduction

The syndrome known under the acronym DRESS (Drug Reaction with Eosinophilia and Systemic Symptoms), also called DIHS (Drug-Induced Hypersensitivity Syndrome), constitutes an adverse drug reaction with systemic involvement. This is a life-threatening clinical-dermatological emergency that presents with a variety of symptoms and signs, including skin rash (especially of the exanthema type), which may be accompanied by fever, lymphadenomegaly, hepatitis, hematological (especially atypical lymphocytes and eosinophilia), renal, cardiac, pulmonary and pancreatic changes.^1^

The term DRESS was initially proposed by Bocquet et al., in 1996, in a review article on the drug hypersensitivity syndrome, which had been previously described under several different terms or synonymies, such as dapsone syndrome, anticonvulsant hypersensitivity syndrome, hydantoin syndrome, and allopurinol hypersensitivity syndrome, depending on the pharmacological agent involved, throughout the 20^th^ century.^2^

The first case description compatible with DRESS was reported by Myers et al., in 1937. A man with gonorrhea, treated with sulfanilamide, in Detroit (USA), presented with a pruritic morbilliform rash, which progressed to exfoliative dermatitis. Subsequently, he developed fever, jaundice, and progressive eosinophilia. In the report, similar cases of exfoliative dermatitis are mentioned, with leukocytosis, hepatitis and eosinophilia in patients treated with arsenical compounds.^3^

In 1938, Merritt & Puttman reported their results on 142 patients with convulsive diseases treated with diphenylhydantoin. Serious adverse events such as purpura and dermatitis were observed in 5% of them, with a 55-year-old male patient developing exfoliative dermatitis 14 days after using the medication, albeit without fever.^4^

In the 1940s, several cases of lymphadenopathy associated with the use of anticonvulsants were described, the first of them accompanied by intense skin rash and fever, due to the use of diphenylhydantoin.^5^

The first fatality with this type of reaction was described in 1946, after the use of tridione, associated with phenylhydantoin, in an adolescent.^6^

The primacy of observing a case without skin rash, although with a variety of symptoms and signs of DRESS syndrome and peripheral eosinophilia, fell to Saltztein et al., in 1958, who described a boy, aged seven, who used ethylphenylhydantoin. The child was hospitalized with cervical lymphadenopathy, seven weeks after the introduction of the anticonvulsant; however, he had previously been treated with metharbital and mephobarbital. There was no skin rash; however, the patient had hepatosplenomegaly and eosinophilia (15%), in addition to fever. Clinical and laboratory changes regressed after four months of drug withdrawal.^7^

In 1950, an adult with epilepsy was described who, after 17 days of sodium diphenylhydantoin use, developed malaise, anorexia, nausea and generalized muscle pain, and subsequently developed jaundice, fever, and a rash that progressed to pruritic exfoliative dermatitis and lymphadenopathy with abdominal pain. The blood count showed lymphocytosis (43%), with 24% atypical lymphocytes and 6% eosinophils which, on the fourth day of hospitalization, developed into 20% peripheral eosinophilia. At the time, the authors observed that, while lymphocyte atypia decreased in the blood count, the eosinophilia progressively increased, configuring a characteristic hematological evolution of what is currently called DRESS syndrome.^8^

In 1949, Lowe and Smith were the first to publish what would be called dapsone hypersensitivity syndrome or sulfone syndrome, when they observed a 2% incidence of development of exfoliative dermatitis in patients receiving dapsone to treat leprosy.^9^ In 1951, sulfone syndrome was described as a mononucleosis-like syndrome characterized by the occurrence of exfoliative dermatitis, hepatomegaly, jaundice, splenomegaly, lymphadenopathy and a predominance of mononuclear cells in the peripheral blood, in patients treated for less than two months with the drug. Of 153 cases of leprosy treated with dapsone in Nigeria in 1951, seven cases (4.5%) of sulfone syndrome were observed within two months of starting treatment.^10^

Over the decades, a variety of medications involved in triggering the DRESS syndrome were identified, especially antiepileptic drugs, antibiotics, antituberculosis drugs, and non-steroidal anti-inflammatory drugs (NSAIDs), among others. Furthermore, the letter “R” for “Reaction” in the acronym DRESS was, by many authors, replaced by “Rash”, which may be inadequate, since, occasionally, visceral involvement without cutaneous symptoms can be observed.^11^, ^12^

This is a narrative review using the terms: “DRESS syndrome”, “anticonvulsant syndrome”, “hydantoin syndrome”, “DRESS” and “DRESS syndrome”, “Drug-Induced Hypersensitivity Syndrome”, “Dapsone Syndrome”, “DRESS” and “Delayed-Hypersensitivity Syndrome”, carried out from 01/01/2011 to 11/01/2023, using the “full text” data filter, in the following databases: PubMed/Medline and LILACS, resulting in 2,635 articles, of which 85 were selected to constitute this review.

## Epidemiology

As its report is not compulsory, the real incidence of DRESS is not known. Similarly, the occurrence of DRESS depends on the type of medication and the patient immune status; many cases are underdiagnosed or go untreated and are not registered in pharmacovigilance systems. The unpredictability of the reaction in association with its potential severity justifies the extensive medical knowledge about the disease.

It is estimated that one case of DRESS occurs for every 10,000 drug exposures, particularly anticonvulsants such as carbamazepine.^11^, ^12^ Among antituberculosis drug users, DRESS occurs in 1.2% of the patients.^13^ In a retrospective study of 1,253 adult patients receiving antituberculosis drugs between 2006 and 2010, 15 cases (1.2%) of DRESS were observed, in patients with a mean age of 65 years (48‒74), 53.3% men, with ethambutol being the most used agent (53.5%), followed by rifampicin (26.5%), pyrazinamide (20.0%), streptomycin (13.3%) and isoniazid (6.7%), with a mean latency period of 42 days for developing symptoms and signs of DRESS.^14^

There is no clear gender predilection among cases of DRESS syndrome; however, Kardaum et al. described a slight predominance of cases in women (male: female ratio 0.80).^15^

According to information from the RegiSCAR16 study group, in 2011, there was a higher frequency of a previous history of rheumatological disease or vascular connective tissue disease in 8.5% of patients with DRESS. As for Japanese patients, approximately half of them showed a history of influenza-like infection within a period of one month preceding DRESS.^12^

The specific mortality rate for DRESS varies from 3% to 20%, representing a dermatological emergency.^15^, ^16^, ^17^ Approximately 94% of the patients require hospitalization, with an estimated economic cost to the American healthcare system of at least US$17,000 per patient.^16^, ^18^ In the study by Wolfson et al. which analyzed data from hospital allergy records in the USA between 1980 and 2016, the authors concluded that the prevalence of DRESS was 2.18 cases for every 100,000 hospitalized patients.^18^

## Pathogenesis

The pathogenic mechanisms involved in the occurrence of DRESS are still controversial. This is probably related to the variety of drugs implicated in triggering this adverse reaction and the interaction of elements such as genetic susceptibility, drug-metabolizing capacity, interactions with previous infections, or activation of latent viruses.^12^, ^17^

More than 60 medications have been associated with DRESS.^11^ The most often implicated are aromatic anticonvulsants (phenytoin, carbamazepine, oxcarbazepine, phenobarbital, primidone, lamotrigine); sulfonamides; sulfones (dapsone); NSAIDs (piroxicam, ibuprofen, diclofenac, paracetamol); beta-lactam antibiotics, vancomycin and minocycline; allopurinol and antiretrovirals. Antibiotics such as amoxicillin can cause DRESS; however, in most cases, this medication acts as an aggravating factor in DRESS induced by other medications. Table 1 describes the main medications listed as potential triggers for DRESS.^11^, ^17^, ^19^, ^20^

**Table 1:** Medications associated with triggering of DRESS.

Pharmacological Groups			Drugs
Anticonvulsants		Aromatic	Phenytoin^a^, carbamazepine^a^, primidona, oxcarbazepine, phenobarbital^a^, lamotrigine^a^
		Non-aromatic	Lamotrigine, ethosuximide, sodium valproate or valproic acid, zonisamide, mexiletine, levetiracetam^a^, benzodiazepines, gabapentin
Antibiotics			Sulfonamides^a^, sulfamethoxazole-trimethoprim, sulfone (dapsone)^a^, sulfasalazine^a^, salazosulfapiridine^a^, rifampicin^a^, etambutol, pyrazinamide, isoniazid, streptomycin, minocycline^a^, azithromycin, ampicillin, ampicillin/sulbactam, ceftriaxone, cefepime, clindamycin, vancomycin^a^, spiramycine, metronidazole, amoxicillin, amoxicillin/clavulanate, piperacillin-tazobactam, augmentin, levofloxacin, teicoplanin, benznidazole^a^, linezolid
Angiotensin Converting Enzyme Inhibitors			Enalapril, captopril
Antidepressants – serotonin reuptake inhibitors			Fluoxetine, bupropion
Classic Antidepressants			Desipramine, amitriptyline, clomipramine
Antifungals			Terbinafine, voriconazole
Antipsychotics			Olanzapine, quetiapine
Antimalarials			Hydroxychloroquine
Nonsteroidal anti-inflammatory drugs			Acetylsalicylic acid (AAS), celecoxib, diclofenac, ibuprofen, piroxicam, dipyrone (metamizole), phenylbutazone, dexketoprofen, codeine
Antivirals	Protease inhibitor		Telaprevir, boceprevir
	HIV integrase inhibitors		Abacavir^a^; raltegravir
	Non-nucleoside reverse transcriptase inhibitors		Nevirapine^a^; zalcitabine
	Reverse transcriptase inhibitors		Abacavir
Immunomodulators			Lenalidomide; leflunomide; thalidomide; mesalazine; teriflunomide
Kinase inhibitors			Vemurafenib; sorafenib; imatinib; pazopanib
Xanthine oxidase inhibitors			Allopurinol^a^; febuxostat
Immune Checkpoint Inhibitors			Nivolumab; ipilimumab
Antineoplastics			Gemcitabine, azathioprine, chlorambucil, amlodipine, diltiazem, spironolactone, vismodegib, efalizumab, bortezomib, alectinib
Other categories			Omeprazole, Propylthiouracil, temozolomide, furosemide, atorvastatin, Traditional Chinese Medicine, quinine, mexiletine, esomeprazole, strontium ranelate, erythropoietin alfa, ranitidine, thiamine, cyanamide, B12 complex, sitagliptin, tribenoside, iodinated radiocontrasts, rivaroxaban, AstraZeneca vaccine against COVID-19, gadolinium contrast, trimetazidine, sildenafil, denosumab, daclizumab, pirfenidone, metformin

a Drugs most frequently listed in the literature.

The understanding of the pathogenesis of DRESS has been increased over decades and has been improved as knowledge advances in genetics, metabolic pathways, the immune system and the reactivation of latent viruses in the body. Currently, DRESS is considered a serious, idiosyncratic adverse drug reaction, mediated especially by T-cells, classified as a type IVb hypersensitivity reaction (sometimes IVc), according to the Gel & Coombs classification, expanded by Pichler.^21^ Thus, the current concept is that DRESS is the result of the complex interaction between exposure to medications (or immunizing vaccines, or immunobiologicals), genetic predisposition, and reactivation of latent viruses, particularly from the *Herpesviridae* family.^20^

**Figure 1:**
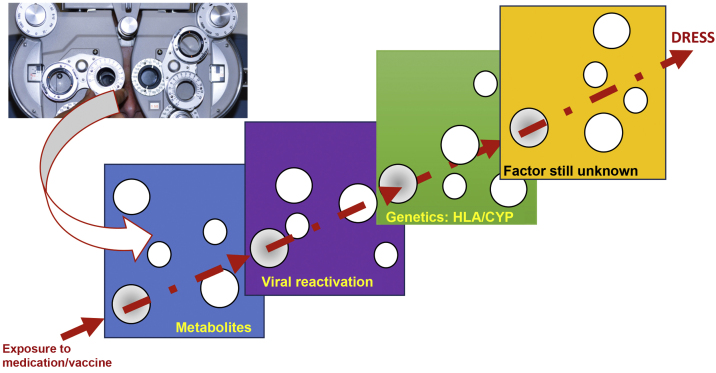
Model of the conjunction of risk factors for the occurrence of a DRESS event, in a model similar to the perfect meeting of risk factors, such as a hole in the same position in different slices of Swiss cheese, or in analogy to the meeting of perfect lenses, during refraction in an ophthalmological examination. The drug, or vaccine, needs to find a metabolic system in humans that generates a certain metabolic intermediate, which generally accumulates in excess, in the same patient, where this metabolite stimulates the proliferation of the monocytic-macrophage system, which leads to the activation of latent viruses incorporated into the host DNA. This human host must have certain genetic characteristics (HLA or cytochrome P450-CYP gain- or loss-of-function mutations), which lead to a specific type of immune response capable of generating the clinical response of DRESS.

The reason why some individuals develop this adverse reaction, while others do not, despite being exposed to the same medication, seems to result from the cumulative effect of the conjunction of risks, which are aligned through a single successive causal model, where the final result, in a straight line, is DRESS (Fig. 1).^20^

The exact mechanism of the adverse reaction called DRESS is complex and has not yet been completely clarified.^11^, ^19^, ^20^ However, there are three essential components that must be considered in its pathogenesis: (i) Presence of genetic susceptibility in relation to certain alleles of the Human Leukocyte Antigen (HLA); (ii) Occurrence of changes in drug metabolism pathways, especially in relation to aromatic anticonvulsants, and (iii) Reactivation of viruses from the *Herpesviridae* family, particularly Human Herpes Virus 6 (HHV6), which leads to a T lymphocyte-mediated inflammatory response that results in varied tissue damage.^11^

### HLA system

HLA alleles constitute one of the most relevant risk factors for the development of DRESS. Despite their importance, it should be noted that risk alleles are specific to certain drugs, in specific ethnicities, suggesting that their occurrence in the host is necessary, but not sufficient in itself to promote a complete immune response.^11^, ^19^

One example of a pharmacogenomic study, where an HLA haplotype is associated with susceptibility to DRESS, is the association between the use of abacavir, in Antiretroviral Therapy (ART), and the increased risk of developing DRESS, if the patient is Caucasian or African-American and a carrier of HLA-B*5701. In turn, the same haplotype in the Han Chinese ethnic group, constitutes a high-risk factor for the occurrence, with the use of allopurinol, of other reactions such as Stevens-Johnson Syndrome (SJS), Toxic Epidermal Necrolysis (TEN) and DRESS syndrome.^11^ Thus, the same HLA alleles can cause different adverse reactions with the same medication, in different ethnicities, or with different medications, in the same population (Table 2).

**Table 2:** Association of HLA alleles, according to drug and ethnicity.

Drug	HLA Allele	Ethnicity
Abacavir^a^	B*57:01	Europeans, Africans and North Americans
Allopurinol	B*58:01	Han Chinese, Korean, Taiwanese, Thai
Carbamazepine	A*31:01	Europeans, Chinese, Koreans, Japanese
Dapsone	B*13:01	Chinese, Taiwanese, Thai, Papuan
Phenytoin	A*24:02	Europeans (Spanish)
	B*51:01	Thai
	C*14:02	Thai
	B*15:13	Malaysians
Phenobarbital	A*01:01	Thai
	B*15:02	Han Chinese, Thai
Lamotrigine	B*51:01 e A*24:02	Europeans (Spanish)
Methazolamide	B*59:01	Koreans, Japanese and Han Chinese
Nevirapine^a^	CW*04:01	Han Chinese, Malays, Thai
	Cw*08 e B*14	Italians (Sardinia), Japanese
	B*35:01	Australians
	B*35:05	Asiabs (Thai)
	B*53:01	Australians
	DRB1*01:01	Australians and Caucasians
Piperacillin/Tazobactam	B*62	British Caucasians
Raltegravir	B*53:01	Africans and Hispanics
Salazosulfapyridine	B*13:01	Han Chinese
Sulfametoxazol	B*38	Europeans
Vancomycin	A*32:01	North-Americans and Europeans

a After the medication meaning it does not completely meet the DRESS criteria.

In terms of mechanism, the implicated drug is believed to interact with its specific HLA to form a hapten-HLA complex, which is then presented to T naïve cells (TH0), via a T-Cell Receptor (TCR) to stimulate an immune response.^19^

It is important to highlight that the associations between the risk of a given drug and a specific HLA have a high negative predictive value and moderate to low Positive Predictive Values ​​(PPV). To date, the highest known PPV is approximately 50% for abacavir-induced DRESS cases. In other words, the low PPV of HLA allele associations with exposure to specific medications suggests that additional factors contribute to the triggering and clinical onset of DRESS. Moreover, allele frequencies vary greatly between different ethnicities, making it important that recommendations be made not only based on the drug to be used but also on the ethnic origin of the individual being treated with it.^19^

### Metabolic pathways of medications

**Figure 2:**
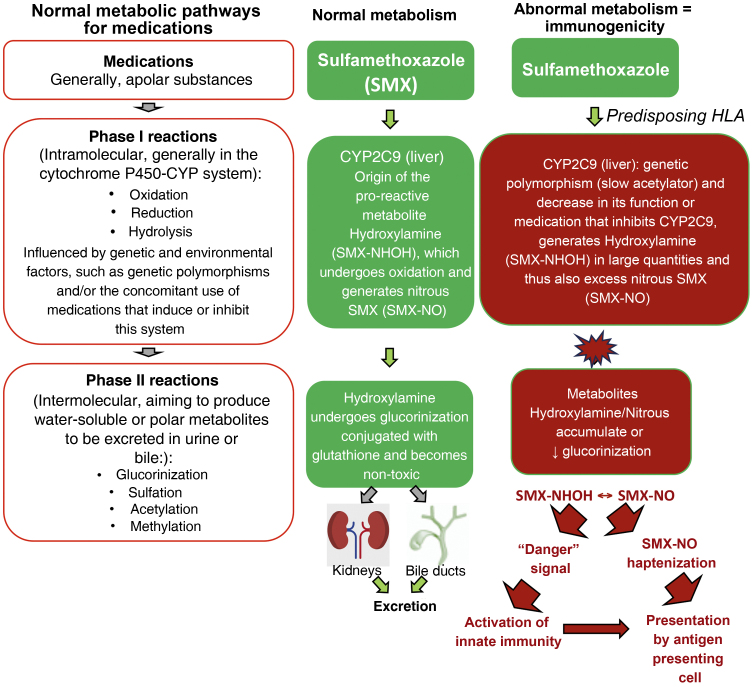
Normal metabolic pathways for drug elimination and an example of the abnormal metabolism of sulfamethoxazole (SMX) in patients with HLA predisposition and loss-of-function (LOF) alterations of metabolic enzymes such as cytochrome P450 (CYP) and/or phase II (intermolecular) reactions such as defects in the glucorinization generating excess hydroxylamine/nitrous metabolites that can produce “danger” signals to the immune system, with direct cell damage and activation of innate immunity and/or presented as haptens to antigen-presenting cells.

Mutations in several drug detoxification enzymes have been associated to cases of DRESS. For example, aromatic anticonvulsants are metabolized by CYP (cytochrome P450 - phase I of drug metabolism) into arene-oxide metabolites, normally detoxified in the conjugation pathways (phase II of drug metabolism) into inactive metabolites by epoxide hydrolase or glutathione transferase. In the case of deficiency of these enzymes (due to gene polymorphisms, or acquired by events such as viral infections, as in HIV infection), these accumulated arene-oxide intermediates act as haptens, stimulating the immune response or binding to tissue macromolecules and causing direct cell damage. (Fig. 2).^19^

It has been demonstrated that patients with DRESS related to sulfonamides have, in many cases, a high incidence of slow acetylators carrier status (low N-acetylation), which determines N-acetyl-transferase deficiency, resulting in the formation of excessive amounts of hydroxylamine metabolites, which reach toxic levels and are capable of causing cell damage and immune activation.^19^

In Taiwanese, Japanese and Malaysian populations, a CYP2C9*3 gene variant (capable of reducing phenytoin excretion) is frequently found and is especially associated with DRESS triggered by this medication use. It also seems that the synergism between predisposition to a certain HLA allele and polymorphism in drug metabolism is necessary to trigger DRESS, as in the case of patients with increased risk of reaction who are exposed to allopurinol, express HLA-B*58:01 and have decreased renal function, which leads to an increase in serum levels of allopurinol or its toxic metabolites.^19^

### Immunological pathways

There are several models by which the drug can stimulate the immune response and cause DRESS: (i) The hapten model, where antigen-presenting cells (APCs) and their HLA have a neoantigen to the TCR of a T naïve cell through a strong covalent bond; (ii) The proposed model of “pharmacological-interaction (PI concept)” where the drug or metabolite+endogenous peptide occupies the space between the HLA of the APC and the TCR of a T naïve lymphocyte with a non-covalent, temporary bond and interactions occur regardless of drug processing by the APC, directly inducing T-cell activation, or (iii) Through the “altered self-peptide repertoire” model, in which a drug linked to an altered endogenous peptide binds, in turn, instead, to an HLA or TCR, changing the binding conformation and is recognized as immunogenic, without the processing of the medication.^22^

Through cell-to-cell interactions, APCs are believed to activate drug-specific T cells, with the help of co-stimulatory molecules such as OX40, which prevent T cells from being inhibited by regulatory T cells (Tregs).^23^ Activated Th2 cells release cytokines, including IL-4, IL-13, and IL-5, inducing eosinophilia in tissues and peripheral blood.^23^

Furthermore, eotaxin produced by keratinocytes and TARC (thymus and activation-regulated chemokine) produced by dendritic cells (DC), promote the harmful accumulation of eosinophils in the skin and other internal organs. IL-33, which is produced by macrophages in the skin of patients with DRESS, also attracts type 2 lymphocytes of innate immunity (ILC2 ‒ innate lymphoid cells type 2), through the ST2 receptor, promoting tissue eosinophilia.^24^

Th2 cells interact with dermal dendritic cells through the costimulatory molecules CD40L and CD40. The costimulatory receptor OX40 (CD134) is a member of the tumor necrosis factor receptor superfamily (TNFRSF), which is expressed on activated CD4+ and CD8+ T cells, neutrophils, and natural killer (NK) cells.^23^ OX40 has relevant costimulatory functions in the activation, survival and expansion of CD4+ and CD8+ T cells, and its binding with the ligand (OX40L) makes them less responsive to inhibitory signals from Tregs.^23^ In DRESS, peripheral blood mononuclear cells (PBMCs) also express OX40L, and the percentage of CD4+ T cells expressing OX40 correlates with the pattern of Th2-mediated immune responses.^25^

Patients with DRESS, in the acute phase of the reaction, show an increase in the number of Tregs and the total CD4+ T cells in the blood, when compared to healthy controls, which is not observed in TEN or other maculopapular drug eruptions. A large proportion of these Tregs have the ability to migrate to the skin, so there is an increase in FOXP3+ T cells in the skin of patients with DRESS when compared to patients who develop SJS/TEN. In the acute phase of DRESS, Tregs increase in number and subsequently decline during resolution.^25^

CD8+ T cells are attracted to the dermis and cause keratinocyte apoptosis by releasing granulolysins. Together with elevated levels of pro-inflammatory cytokines, including interferon-gamma (INF-γ), tumor necrosis factor (TNF), IL-6, and IL-15, these cytokines promote the systemic inflammation characterized as DRESS.^25^

DRESS is characterized by a variety of hematological abnormalities that include leukocytosis, atypical circulating lymphocytes, and peripheral eosinophilia. While eosinophilia is not found universally in all patients, a Th2-mediated reaction is observed in tissues, with an abundance of IL-4, IL13, and IL-5.^26^ Polarized Th2 lymphocytes are recruited by TARC/CCL17 to inflammation sites, but other cytokines are also found in addition to those already mentioned, IL-2 and granulolysins. In the late phase of DRESS, there is an increase in the number of CD*+ T lymphocytes and Th1 cells, often concomitantly with disease exacerbation, worsening of hepatitis, and an increase in antibodies against HHV6.^19^

The reactivation of latent viral infections during delayed-type drug hypersensitivity reactions is considered a specific characteristic of DRESS syndrome. The most prevalent reactivated infection is HHV6, which can be found in peripheral blood, skin, lymph nodes and kidney tissue. Moreover, subsequently, there may be reactivation of other viruses from the *Herpesviridae* family, such as HHV7, the Epstein-Barr virus (EBV), and, finally, cytomegalovirus (CMV).^27^ HHV6 reactivation requires immunosuppression, which is demonstrated by the decrease in serum levels of immunoglobulins, including IgG, IgM and IgA, in addition to circulating B cells at the onset of DRESS syndrome.^24^

The depletion in circulation and in DRESS skin lesions of pro-inflammatory CD14dimCD16+ monocytes (pMOs) capable of mediating antiviral functions, together with the significant expansion of CD4+FOXP3+ Treg cells in the acute phase of DRESS, aiming to contain the activation of drug-specific effector T cells (Teffs), could play a relevant role in inhibiting antiviral T lymphocytes and favoring the reactivation of latent viruses.^12^, ^24^

**Figure 3:**
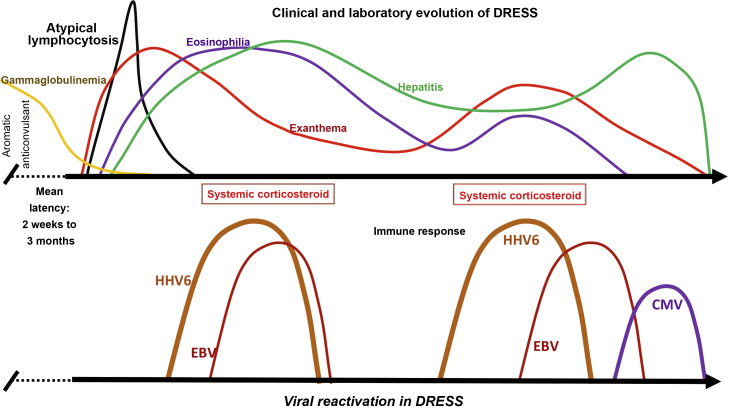
Clinical and laboratory evolution of DRESS. Sequential reactivation of the *Herpesviridae* family, after progressive hypogammaglobulinemia, previously classically demonstrated in patients using aromatic anticonvulsants, contributing to the resurgence of clinical and laboratory manifestations, even after withdrawal of the drug that triggered DRESS.

In some patients with DRESS, HH6 and HH7, EBV, and CMV are detected in the blood, and in some human tissues, in addition to, in some cases, Varicella zoster virus (VZV). Between 76% and 80% of patients with DRESS test positive for HHV6, HHV7, or EBV, suggesting that viral reactivation contributes to the course of the disease.^16^ This viral reactivation is generally detected between two and four weeks after DRESS onset, and may be associated with symptom recurrence after the initial improvement demonstrated by the patient (Fig. 3).

Recently, reactivation of the JAK-STAT signaling pathway was demonstrated in a patient with refractory DRESS syndrome, triggered by the use of trimethoprim-sulfamethoxazole. Among so many type 2 inflammatory response circuits in DRESS, IL-5 possibly plays a critical role in disease pathogenesis. Approximately 30% of the patients with DRESS syndrome develop complications, including infections, and inflammatory and autoimmune diseases.^16^, ^28^, ^29^

Although the contribution of the *Herpesviridae* family to the pathogenesis of DRESS is not yet definitive, viral reactivation can occur in the absence of immunosuppressive therapies and the emergence of virus-specific CD8+ T lymphocytes suggests that herpes virus reactivation is an integral component of the DRESS pathogenic process. In summary, in DRESS, T cells in the skin and blood demonstrate non-clonal proliferation, distinct expression of chemokine receptors, co-stimulatory molecules, and positive gene regulation in the JAK-STAT signaling pathway.^28^, ^29^

## Clinical manifestations

DRESS syndrome is characterized by the gradual involvement of multiple organs, which may include skin, hematological systems, and solid organs.^19^ Clinical manifestations are usually perceived two weeks to two months after starting a medication.^22^

DRESS begins with prodromes of flu-like malaise, pharyngitis, fever, and lymphadenopathy.^30^ The evolution can be slow and with the association of several symptoms, with fever (≥ 38.5 °C) occurring in 75%‒100% of patients and generally preceding the rash by several days.^15^, ^16^, ^19^

Antibiotics or radiocontrast media can trigger the reaction within approximately 14 days of exposure, while antiepileptics and allopurinol tend to have longer latency periods and may be related to more severe forms of the disease.^26^, ^31^

### Skin manifestations

Although the dermatological manifestations are varied and affect more than 50% of the body surface, the most characteristic lesions in the initial phase are periorbital and facial edema (76% of cases) with micropustules.^15^, ^17^, ^26^, ^32^^,^^33^

**Figure 4:**
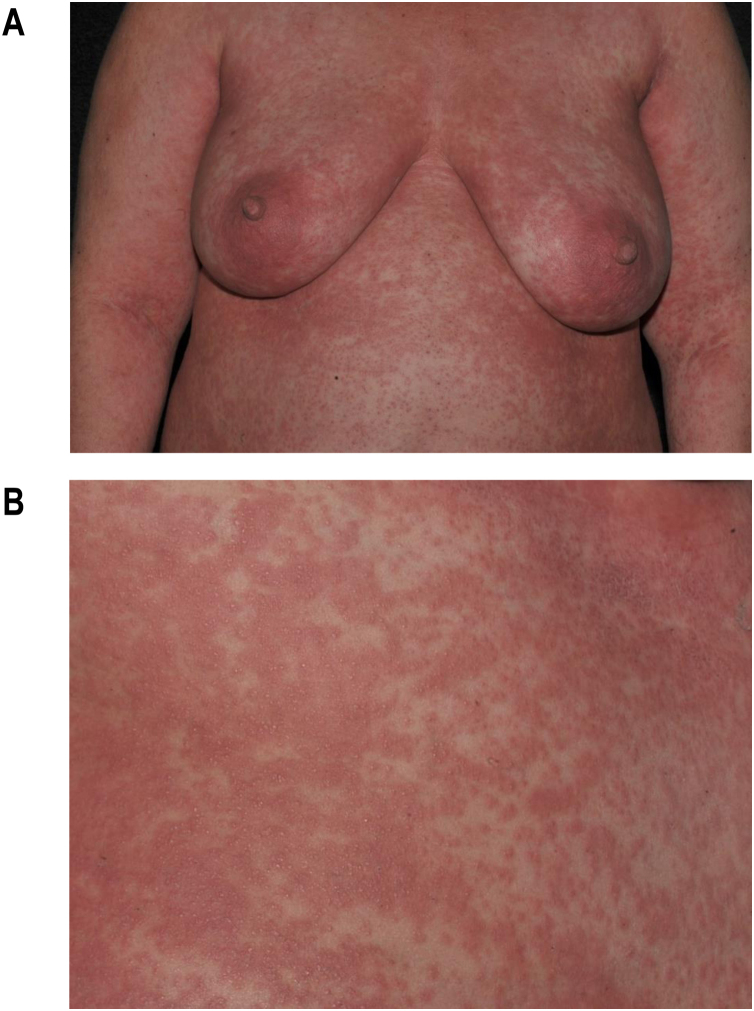
Maculopapular rash in DRESS. (A) Extensive trunk involvement. (B) Detail of the lesions: erythematous papules that converge into plaques.

Maculopapular exanthema is the most common clinical sign, presenting in 99%‒100% of patients, sparing the palms and soles, with craniocaudal progression (Fig. 4).

Pruritus is common and there may be burning pain.^34^ Mucosal involvement can be seen in up to 56% of patients; however, it is typically mild and non-hemorrhagic, differentiating it from SJS/TEN.^15^, ^33^ Children are more prone to morbilliform rash, fever, and lymphadenopathy, but less likely to have facial edema.^35^

The erythrodermic form of DRESS is considered rare, while the morbilliform rash affects more than 80% of the patients. Erythroderma occurs in only 7.4% of cases.^2^, ^36^

Vesicular and bullous lesions can be induced by dermal edema in DRESS; as there is no extensive necrosis in the epidermis, the bullae are different from those seen in SJS/TEN, but the differential diagnosis is potentially difficult and histopathological examination is indicated.^37^, ^38^

**Figure 5:**
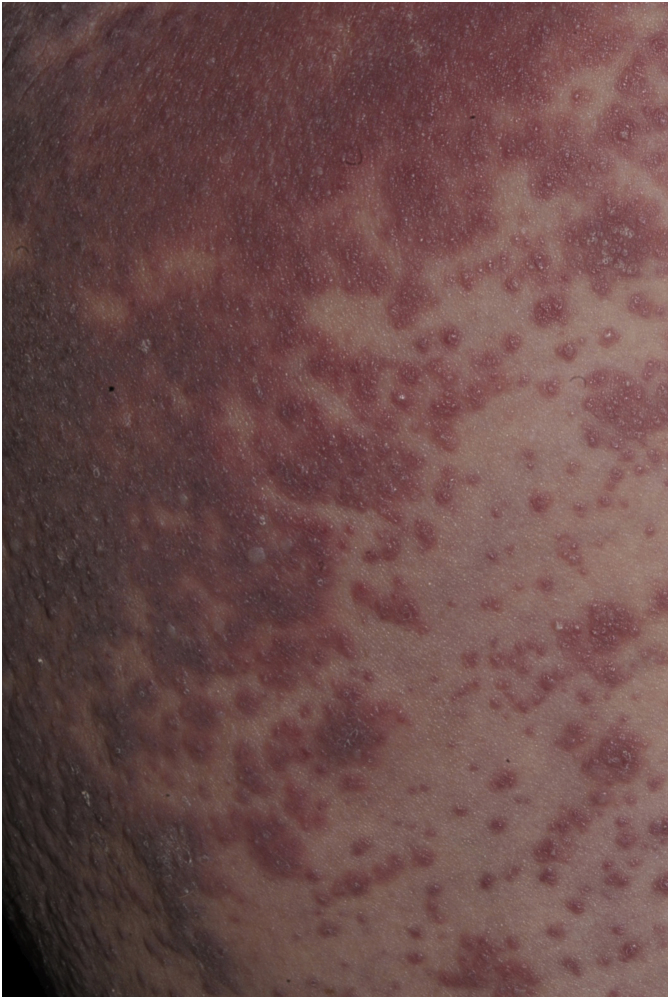
Atypical form of DRESS. Erythematous-edematous plaques with sterile vesicles and pustules.

Patients with DRESS may also have sterile, perifollicular pustules, as well as small non-follicular pustules, simulating acute generalized exanthematous pustulosis (AGEP; Fig. 5).^2^

### Extracutaneous manifestations

The alarm signal for DRESS should be systemic involvement with affected internal organs, which can occur in up to 91% of patients, with liver involvement being the most common, followed by kidneys and lungs.^15^ Lymphadenopathy occurs in 50%‒75% of patients.^34^

Liver involvement due to DRESS occurs in up to 97% of cases, so it is the most common visceral manifestation. The most common finding is elevated liver enzyme levels (cholestatic, mixed and hepatocellular). In some cases, fulminant liver failure may occur. Hepatitis is typically anicteric and elevated liver enzymes may take months to resolve completely.^15^, ^30^, ^33^

The second most frequently affected organ is the kidney, ranging from mild acute kidney injury to severe interstitial nephritis, resulting in permanent end-stage kidney disease. Patients who are most at risk of renal failure from DRESS are elderly individuals, those with pre-existing renal disease and DRESS caused by allopurinol.^15^, ^30^, ^39^

The lung is the third most frequently affected organ, with interstitial pneumonitis as the most common manifestation.^39^ The association with minocycline may lead to a higher incidence of pneumonitis.^40^

Cardiac involvement typically manifests as myocarditis or pericarditis and, on average, 70 days after the initial symptoms. The most common signs and symptoms of cardiac DRESS are dyspnea, cardiogenic shock, chest pain, and tachycardia.^41^

Patients with DRESS may develop, less frequently, pancreatitis, colitis, cholangitis, encephalitis/meningoencephalitis, hemophagocytic syndrome and thyroiditis. Moreover, there have been reports of spleen, stomach and central nervous system involvement.^24^, ^30^, ^40^

Pediatric cases do not seem to differ from adult ones. The mean time between medication intake and clinical manifestations was 18.9 days, and DRESS recurrence occurred in 4.8% of cases. Eleven percent of children may experience long-term sequelae, such as autoimmune diseases (most commonly hypothyroidism, 3.8%).^27^, ^42^

## Histopathological changes

The histopathology of DRESS varies depending on the stage of the disease and clinical manifestations and, since there are no pathognomonic findings, correlation with clinical and laboratory criteria is necessary for a definitive diagnosis. However, histopathology is recommended to differentiate it from other dermatoses.^43^

**Figure 6:**
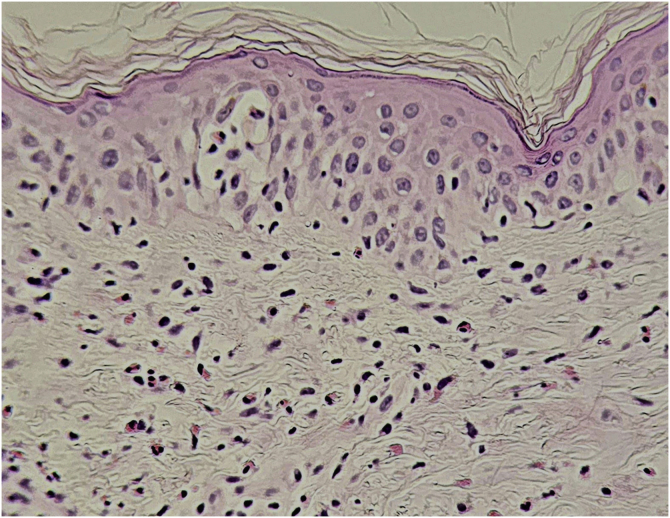
DRESS (Hematoxylin & eosin, ×400). Vacuolar interface dermatitis, mild spongiosis, lymphocyte exocytosis. There is diffuse lymphocytic infiltration in the dermis, with frequent eosinophils.

Histopathological findings can be polymorphous, with basal layer vacuolation, lymphocyte exocytosis, keratinocyte necrosis, perivascular and/or diffuse lymphocytic infiltrate, with frequent eosinophils (Fig. 6). Dermal edema and erythrocyte extravasation may be present, as well as spongiosis, a pseudolymphomatous reaction, and focal epidermal necrosis.^43^, ^44^

## Laboratory alterations

Eosinophilia, in varying degrees, is one of the characteristics of the syndrome, is present in up to 95.5% of cases at some stage of the disease and may persist after the normalization of liver enzymes.^32^, ^45^

Several cytokines are involved in the activation and recruitment of eosinophils; however, it is believed that TARC/CCL17 (thymus and activation-regulated chemokine) levels are higher in DRESS compared to other severe drug reactions and occur in the acute phase of the disease, inducing the eosinophilia.^11^, ^32^, ^45^ TARC is a potent chemokine that recruits eosinophils and increases cutaneous lymphocyte attraction by targeting CD4+ and CD8+ T cells to the skin, promoting the production of IL-5 and eotaxin.^11^, ^32^, ^45^ IL-5, in turn, is a crucial cytokine for eosinophil development, activation and survival.^19^, ^22^

Eosinophil counts and TARC levels are associated with greater disease severity, involvement of multiple organs and systems, as well as the intensity of the immune response triggered by activated T cells in response to exposure to the medication, as well as viral reactivation from the human herpesvirus family, especially HHV6.^11^, ^22^, ^45^

Other hematological alterations are observed in DRESS. Leukocytosis with atypical lymphocytes of varying degrees of intensity are uniquely characteristic of the early phase of the disease, although leukopenia may occasionally precede the leukocytosis.^19^, ^22^ Leukocytosis with early neutrophilia and late monocytosis is, after eosinophilia, the most common hematological alteration, followed by atypical lymphocytosis in 27%–67% of the patients.^15^, ^44^ Other less frequent findings include lymphopenia, leukopenia, thrombocytopenia, thrombocytosis, and pancytopenia, which are associated with a more severe prognosis.^15^, ^33^, ^46^

Leukocytosis is largely due to the increase in lymphocytes caused by the massive activation of CD4+ and CD8+ lymphocytes, initially by the medication and after its withdrawal, by the reactivation of latent viral infections.^19^, ^23^ Atypical lymphocytosis is a nonspecific response to a probable antigenic stimulus (virus, drug, graft-versus-host reaction), where there is rapid production and early release of immature lymphocytes, which are subsequently removed from circulation before mitosis.^47^ Therefore, a high percentage of atypical lymphocytes may be a reflection of more intense stimulation by the antigen (drug and/or infection).^23^, ^47^ Leukocytosis can also be induced by corticosteroid therapy during treatment.

In the initial stages of DRESS (first 10 days), there is a decrease in the count of NK cells and B cells, in addition to an increase in monocytes and Treg cells. In the subacute phase, Treg cells progressively lose their function after clinical resolution, with their counts returning to baseline levels.^32^, ^45^

Laboratory inflammation markers include elevated levels of C-reactive protein (CRP) and leukocyte counts. CRP is produced by the liver in response to increased IL-6 levels and has pro-inflammatory effects when mediating the humoral and effector cell pathways of the innate immune system. However, nonbacterial inflammation can cause an equally marked elevation in CRP levels. A more specific marker of bacterial infection is procalcitonin (PCT) and its value correlates with infection severity.

CRP and PCT values ​​were found to be elevated in a retrospective observational study of 94 possible, probable and confirmed cases of DRESS syndrome, even when concomitant infection was excluded. CRP values ​​were significantly higher in patients with possible additional causes of inflammation, such as infection. Additionally, a PCT value above the normal cutoff, highly suggestive of bacterial infection, may simply result from the inflammatory state associated with DRESS syndrome. Evaluating CRP and PCT values ​​in light of these results can help clinicians differentiate between cases of DRESS syndrome with and without concomitant infection, or other causes of inflammation. This can further help in decision-making about the best treatment plan in individual cases.^48^

The elevation of hepatocellular enzyme levels occurs in up to 70% of patients with DRESS in the acute phase and can vary from two to more than five times their normal levels. Its normalization occurs both when the disease improves and after massive liver necrosis, which requires careful and constant monitoring. The development of severe hepatitis with jaundice increases the risk of mortality. These severe cases also lead to an increase in prothrombin time and subsequent hypoalbuminemia.^11^, ^24^, ^32^

A variable degree of renal involvement may be observed, with increased urea, creatinine and proteinuria levels. When these levels are 1.5 times above the normal value, they should be carefully monitored.^24^ Creatinine levels can reach up to three times the normal ones, compatible with acute renal failure in the most severe cases.

Overall, DRESS-specific mortality depends on both the degree of renal and liver involvement.^24^ The involvement of other organs and systems should guide the alterations observed in other complementary exams. Table 3 lists supporting laboratory tests to be requested in patients with suspected DRESS.

**Table 3:** Suggested laboratory tests for a patient with suspected DRESS according to the Spanish disease management consensus.^24^

Blood count with evaluation of atypical lymphocytes
Inflammatory markers:
C-Reactive Protein (CRP)
Procalcitonin (PCT)
Liver tests (periodic, if there is liver involvement):
Aspartate Aminotransferase (AST)
Alanine Aminotransferase (ALT)
Gamma-glutamyl transferase (GGT)
Alkaline Phosphatase
Total bilirubin and fractions
Prothrombin Time (INR)
Renal Function (periodic, if there is renal involvement):
Serum creatinine
Serum urea
Urine albumin/creatinine ratio or protein/creatinine ratio, urinary sediment, proteins and cells in urine.
Proteinogram, serum immunoglobulins and serology:
Serologies (IgM and IgG) for herpes viruses and polymerase chain reaction (PCR) for HHV-6, HHV-7, EBV and CMV viruses.
Others:
Lactic dehydrogenase (LDH)
Lipase, amylase
Creatine phosphokinase (CPK)
Troponin I
Blood electrolytes: sodium, potassium
Exclusion of differential diagnoses:
Serology for Mycoplasma, Chlamydia, HAV, HBV, HCV, Parvovirus B19, herpes simplex virus 1 and 2 (Kaposi's varicelliform eruption)
Blood cultures
Antinuclear Antibodies

## Diagnostic criteria

Given the clinical complexity and heterogeneity of manifestations, as well as the possibility of overlap with other diseases, several systems of diagnostic criteria have already been proposed for DRESS. The most used criteria are those of Bocquet et al., the European RegiSCAR (Registry of Severe Cutaneous Adverse Reactions), and the Japanese version of SCAR (Severe Cutaneous Adverse Reaction), the J-SCAR. The reactivation of viruses from the *Herpesviridae* family (HHV-6, HHV-7, CMV and EBV) is not considered in the first two criteria, only in J-SCAR.^2^, ^15^, ^32^, ^49^

Bocquet's criteria (1996) are the easiest to apply, as they comprise the presence of three parameters: i) Skin lesions; ii) Blood eosinophilia (≥ 1.5 × 103/µL) or presence of atypical lymphocytes, and iii) Systemic involvement, including lymphadenopathy (≥ 2 cm in diameter), hepatitis (elevation of transaminases greater than twice the normal limit), interstitial nephritis, interstitial pneumonia, or carditis.^2^ However, this system is less sensitive in diagnosing less exuberant cases when compared to RegiSCAR. Kim et al. proposed that these two systems be used in a complementary way.^49^

The RegiSCAR criteria system is the most detailed one (Table 4), considering those with a score between two and three probable cases of DRESS; possible cases are those with four to five points; and a definitive diagnosis is established when the score is above five.^15^ The RegiSCAR system is the most frequently used, mainly in academia, and is recommended for use in clinical routine by the Spanish guidelines for the diagnosis of DRESS.^24^, ^50^

**Table 4:** Diagnostic criteria for DRESS: RegiSCAR.^15^, ^24^

Score	-1	0	1	2	Min	Max
Fever ≥ 38.5 (trunk) or >38 °C (axillary)	N	Y			-1	0
Lymph node enlargement (>1 cm in size, at least in two places)		N/U	Yes		0	1
Eosinophilia		N/U	700‒1499 μL	≥1500 μL	0	2
			10‒19.9% (if there is leukopenia)	≥20% (if there is leukopenia)		
Atypical lymphocytes		N/U	Yes		0	1
Skin involvement					-2	2
- Extensive rash (% of the body surface area)		N/U	>50%			
- Rash suggestive of DRESS (≥2 of facial edema, purpura, infiltration, desquamation)	N	U				
- Skin histopathology suggestive of DRESS	N	Y/U	Yes			
Systemic involvement (liver/kidneys/lungs/heart muscle/pancreas/others)		N/U	Y/Y/Y/Y/Y/Y		0	2
Resolution in >15 days	N	Y			-1	0
Assessment of other possible etiologies:			Yes (no [+] and, at least 3 [-])		0	1
- Serology for HAV/HBV/HCV/blood culture;						
- Anti-nuclear antibodies/chlamydia/mycoplasma						
Total score	<2, excluded; 2‒3, possible/4‒5, probable;				-4	9
	>5, definitive					

N, No; Y, Yes; U, Unknown.

The J-SCAR defines that, for the diagnosis of typical DRESS, all seven described criteria must be present, including HHV-6 reactivation (Table 5). It should be noted that this system only considers some drugs as potentially causing DRESS. Atypical DIHS cases can be diagnosed when criteria one through five are present.^51^, ^52^

**Table 5:** Diagnostic criteria for DRESS: J-SCAR.^51^ Seven criteria present: typical DRESS, 1 to 5 criteria present: atypical DRESS.

1	Maculopapular rash more than three weeks after starting therapy with certain drugs (carbamazepine, phenytoin, phenobarbital, mexiletine, sulfasalazine, allopurinol and minocycline)
2	Persistence of clinical symptoms two weeks after discontinuation of the causative drug
3	Fever (>38 °C)
4	Liver alterations (ALT >100/L)
5	Leukocyte alterations (at least one):
	a) Leukocytosis (>11 × 10^9^/L)
	b) Atypical lymphocytosis (> 5%)
	c) Eosinophilia (>1.5 × 10^9^/L)
6	Lymphadenopathy
7	HHV-6 reactivation

Recently, a retrospective study showed that RegiSCAR was more accurate in diagnosing DRESS, classifying more than 98% of included cases as “probable” or “definite”, whereas J-SCAR was less sensitive.^49^, ^53^

Furthermore, Sasidharanpillai et al. suggested that reactivation of HHV-6, as well as of CMV, EBV and HHV-7, be used as prognostic rather than diagnostic indicators.^53^

## Identification of the etiological DRESS drug

The identification of the drug causing the reaction is extremely important, not only to interrupt the reaction but to avoid re-exposure to the drug and prevent new episodes of DRESS, allowing guidance on permitted medications after the acute episode of the syndrome.

Over the years, several methods for assessing causality have been developed, including expert judgment or global introspection, operational algorithms, and probabilistic approaches. Unfortunately, there is no specific algorithm for drug causality in DRESS as there is for SJS/TEN.^44^

A detailed clinical history of all medications used, including over-the-counter medications, as well as the consumption of herbal or homeopathic products, and information on drug withdrawal or reintroduction (if available) constitutes the main etiological investigation tool. All medications taken during periods of exposure must be recorded (including the chronology of medication consumption, dose, indication, and clinical evolution after drug withdrawal).^24^, ^44^

Kardaun et al. recommend a series of steps to exclude unlikely medications: (a) Medicines used for more than three months; (b) Medications discontinued more than two weeks before the day the condition started; and (c) Medications started less than three days before the probable day of DRESS onset. Then, medications can be classified as “very likely”, “probable”, “possible” and “undetermined”, ranking them according to the probability reported in the literature.^15^, ^44^

Re-exposure to the drug is not recommended as a strategy to confirm the etiological diagnosis of DRESS; however, specific tests may provide some support in identifying drugs associated with risk of the disease.^54^

### Delayed Patch Test Reading (DPTR)

*In vivo* patch testing is considered a safe test, even when investigating severe and potentially fatal reactions mediated by T cells, such as DRESS. In this context, it is essential to keep in mind that there is always the possibility that DPTR with medications may trigger an exacerbation of the drug-induced rash, although such cases are rare; therefore, the patient must be adequately informed and consent obtained before carrying out the test. It is also important to note that the test may result in other dermatological lesions in addition to papules, vesicles and bullae.^55^

The method consists of occluding the medication, previously diluted in a medium (Vaseline, water), on the skin, together with the vehicle alone, used as control.^55^, ^56^, ^57^ In Europe, Canada and the United States, there are more commercially available preparations for testing drugs, but in Brazil, most substances are tested in accordance with the European guidelines: (i) Testing the commercialized drug diluted at 30% or (ii) Preferably using the pure substance at 10%, unless there is concentration information specific for the substance available in the literature.^55^, ^56^, ^57^

There is no published study in which the drugs suspected of causing DRESS have been tested at different concentrations and vehicles in the DPTR, in a considerable number of patients, and with fully specified results.^55^, ^56^, ^57^ The literature does not provide an indication of the optimal or preferred concentrations and vehicles for DPTR with specific drugs, either.^56^ In intravenous preparations, it is recommended to use the undiluted medication powder for the test. Some substances such as acyclovir and carbamazepine can cause reactions even at low concentrations, and it is recommended to start with much lower concentrations such as 0.1% to 1% and, if the test is negative, increase it to 10%.^15^

DPTR can be applied on the dorsal region, like common patch tests; however, due to its low positivity, it can be performed on skin previously affected in the acute episode of DRESS, as in these sites there is a greater concentration of inflammatory and memory cells.^15^ Similarly to fixed drug eruption, readings can be performed earlier (6 h to 24 h), but it is important to read them between 72-96 hours and another reading can be done after seven days.

De Groot carried out a review of DPTR positivity in patients with DRESS. Among 437 patients with a positive patch test found in the literature, 75 had reactions to two or more drugs. In six cases, the drugs were of the same chemical/structural class. In the other 69 subjects (16% of the total patient population), the medications were of different classes, indicating hypersensitivity to multiple drugs. The most frequently implicated drug classes were anticonvulsants (30%), beta-lactam antibiotics (22%), antituberculosis agents (18%), and non-beta-lactam antibiotics (11%).^58^

DPTR should be performed between six weeks and up to six months after DRESS resolution, and one month after any immunosuppressive treatment. Unfortunately, there is not sufficient evidence about the influence of immunosuppressive drugs on test results.^15^, ^59^, ^60^ A randomized, double-blind study revealed that 20 mg/day of oral prednisone suppressed reactivity in DPTR in patients with nickel sensitivity.^59^ A prospective study reported positive reactions in DPTR in patients taking azathioprine, cyclosporine, mycophenolate mofetil, methotrexate, infliximab, adalimumab, etanercept, and tacrolimus.^60^

The positive predictive value of DPTR in DRESS varies greatly across studies and management guidelines, described as positive in up to 80% of cases in patients with DRESS after the use of carbamazepine, but in only 20% after exposure to phenobarbital.^58^, ^61^

### Intradermal Tests (IDT)

Intradermal tests can be used to identify both immediate and delayed hypersensitivity reactions to medications. Until recently, they were generally considered contraindicated in severe drug reactions; however, some authors perform these tests to improve the accuracy in identifyng the medication that triggers DRESS, considering them potentially useful and safe when performed by specialists.^58^, ^62^

It should be noted that, despite the small doses used, at concentrations lower than in patch testing, serious and even fatal reactions have occurred and European guidelines still contraindicate it.^63^ Therefore, the indication of IDT must be carefully evaluated and is of great importance in reactions caused by antibiotics, due to the possibility of cross-reaction between them.^58^, ^62^

Certain precautions are important when carrying out IDT in patients with DRESS: i) Tests with medications must be carried out in a hospital environment (under supervision) and can only be carried out with commercially available drugs in injectable form; (ii) In severe adverse drug reactions, such as DRESS, IDTs should not be performed with highly suspected medications; (iii) The recommended volume for injection is 0.02 mL; (iv) The diameter of the injection wheal should be measured immediately after the injection (T0) and after 20 minutes (T20); at this moment, the IDT is considered positive if the diameter of the papule is greater than or equal to the diameter of T0+3 mm, and if there is erythema this must be measured; (v) In case of DRESS, IDTs may become positive in late readings, i.e., after 24 h and 48 h.^62^, ^63^

### Lymphocyte Proliferation Test (LPT)

Although scarcely available, *in vitro* diagnostic tests have the advantage of being absolutely safe. They are based on the property of antigen-specific T cells to be activated when stimulated with the nominal antigen in sensitized patients.^24^

They should not be performed before a minimum interval of four to eight weeks after the reaction and at least four weeks after treatment withdrawal with systemic corticosteroids. After the first six months to one year, there may be a decrease in positivity, although the results of tests performed after this time may be positive.^24^, ^64^

The LPT method is based on the evaluation of the proliferation of T lymphocytes labeled with 3H-thymidine, in response to a medication after incubation.^64^ There is great variation in positivity, which is greater for beta-lactam antibiotics. There is yet no complete standardization of the method and it is only available in research centers.^15^, ^24^, ^65^ Recently, the use of cytokines or stimulation of toll-Like receptors (TLR) has been attempted to increase the sensitivity and specificity of these tests.^65^

## Differential diagnoses

Typical forms of DRESS with morbilliform eruption, lymphadenopathy, fever and eosinophilia that begin weeks after starting a medication do not cause diagnostic difficulties.

Some dermatoses can be confused with DRESS, such as Stevens-Johnson syndrome, toxic epidermal necrolysis, extensive erythema multiforme, AGEP, staphylococcal scalded skin syndrome, and febrile drug exanthema.^66^, ^67^, ^68^

The main infectious diseases that can lead to diagnostic confusion are EBV infection (infectious mononucleosis), CMV, hepatitis A, hepatitis B virus and acute HIV infection, COVID-19, Parvovirus B19, *Mycoplasma pneumoniae*, Zika virus, dengue, and secondary syphilis.^69^

The main autoimmune or autoinflammatory diseases to be considered in the differential diagnosis are systemic lupus erythematosus, adult Still's disease, Schnitzler syndrome and Kawasaki disease.

Several hematological diseases are included in the differential diagnosis of DRESS: macrophage activation syndrome/hemophagocytosis, adult T-cell lymphoma/leukemia with an erythrodermic pattern (associated with HTLV-1), pseudolymphomas, and hypereosinophilic syndromes. Reactive lymphadenopathy in DRESS may show histopathological features of angioimmunoblastic T-cell lymphoma, Hodgkin’s disease-like pattern, T-cell lymphoma, necrotizing lymphadenitis (Kikuchi’s disease-like) with or without vasculitis.^70^, ^71^

## Treatment

Early recognition of DRESS and immediate withdrawal of the causal medication are essential elements for therapeutic success and mitigation of systemic damage. Symptomatic treatments such as antipyretics, antihistamines, emollients and topical corticosteroids should be the starting point of patient care.^11^ The use of unnecessary NSAIDs and antibiotics should be avoided when prescribing.^11^

The treatment for DRESS depends on its severity. Milder cases would be those in which, considering the upper normal reference laboratory values: transaminases, especially ALT, do not exceed three times, or alkaline phosphatase does not exceed two times, and bilirubin does not exceed two times. The patient would also not have any organ involvement. In these cases, treatment with topical corticosteroids, emollients and antihistamines can be considered.^11^, ^23^

However, when there is moderate to severe organ involvement, such as transaminases above five times the upper reference value, or an increase above two times in alkaline phosphatase and bilirubin levels, with acute renal involvement (creatinine >2‒2.9 times above baseline or urinary output <0.5 mL/kg/h r > 12 hours, hemophagocytosis, pulmonary or cardiac involvement), treatment must include, in addition to the measures mentioned for mild cases, systemic corticosteroids at a dose of 1 mg/kg/day (prednisone or prednisolone) for two to three weeks with gradual tapering over four to six weeks.^11^, ^23^

In cases where warning signs occur, such as hemophagocytosis, medullary failure, encephalitis, hepatic or respiratory failure or when there is lack of control or contraindication for corticosteroids, cyclosporine can be used at a dose of 4‒5 mg/kg/day for five to seven days (with progressive decrease), intravenous immunoglobulin (1‒2 g/kg/d) for five days or plasmapheresis.^11^, ^23^

DRESS cases that develop into exfoliative erythroderma should be considered more serious due to the risk of fluid and electrolyte imbalance, energy-protein loss, increased risk of infection, and cardiovascular events. Intensive monitoring and support measures are essential to mitigate complications caused by extensive skin involvement.^72^, ^73^, ^74^

Systemic corticosteroids are the therapy of choice in DRESS, at doses equivalent to 1‒1.5 mg/kg/day of prednisone, with gradual improvement in systemic symptoms and laboratory parameters, which can remain altered for more than four weeks. It is essential that the withdrawal of corticosteroid therapy be carried out slowly, requiring one to two months to completely suspend treatment. Abrupt treatment withdrawal, even in the absence of systemic symptoms or laboratory alterations, can lead to DRESS recurrence.^17^, ^75^ In cases with minimal systemic symptoms, symptomatic therapy and medium-potency topical corticosteroids can be used.^76^

Conditions refractory to corticosteroid therapy can be treated with intravenous pulse therapy with methylprednisolone (1 g/d, for three consecutive days). However, despite its immunosuppressive potency, this regimen can lead to CMV reactivation, with greater specific mortality.^77^, ^78^

Other immunosuppressive agents have been proposed as corticosteroid-sparing agents or their substitutes, in cases of contraindication.

Oral cyclosporine promotes rapid control of skin lesions, symptoms, and laboratory parameters. Despite the greater risk of nephrotoxicity associated with the drug, a retrospective study observed a lower recurrence rate when compared with intravenous methylprednisolone, in addition to the shorter therapeutic course.^79^, ^80^

Other described treatments include intravenous immunoglobulin, mepolizumab, dupilumab and plasmapheresis, whit success described in a few case reports.^27^ Specific antiviral treatment (e.g., ganciclovir) is a controversial subject, and although not recommended by some authors,^75^ if signs and symptoms are suggestive and there is confirmation of viral reactivation, specific immunoglobulins and antivirals should be tried.

Systemic involvement must be addressed by a multidisciplinary team, especially with hepatologists, nephrologists and pulmonologists since there is no parallel between cutaneous manifestations and the involvement of other organs.

**Figure 7:**
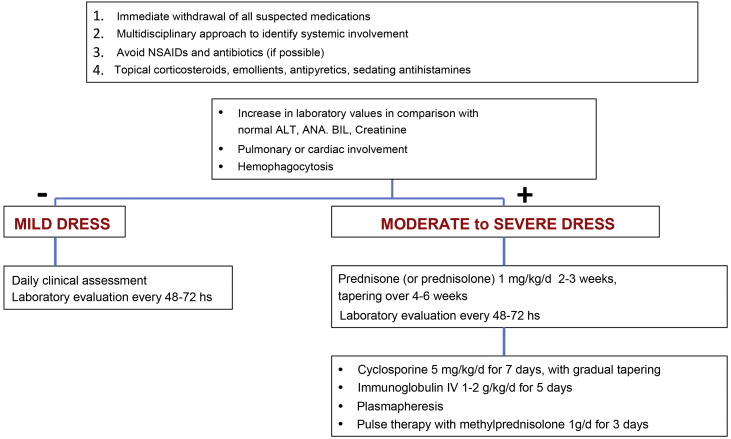
DRESS therapeutic approach flowchart, based on the severity of the condition.

Fig. 7 shows a proposed treatment flowchart according to DRESS severity.^11^, ^75^, ^81^

## Prognosis and final considerations

The prognosis and therapeutic response are more favorable in young people than in the elderly.^27^, ^75^

Re-exposure to the drug involved in DRESS, or to other drugs with an aromatic structure, can cause the condition to recur, so the patient must be informed, in writing, of this risk, especially that linked to aromatic anticonvulsants, allopurinol and sulfonamides.

DRESS cases have variable severity and an unpredictable prognosis. Serious complications, whether or not related to CMV reactivation, constitute a highly likely cause of death.^22^

Elderly patients, with comorbidities, in poor health, in which systemic manifestations are more evident, in whom the causative medication has been perpetuated for a longer time, or in whom specific treatment has been delayed tend to have a worse prognosis and slower recovery.^82^, ^83^, ^84^, ^85^

Systemic autoimmune sequelae can appear months and even years after resolution, such as autoimmune thyroiditis, fulminant type I diabetes, autoimmune hemolytic anemia and alopecia. This strongly suggests the need for long follow-up, even after clinical resolution of the acute phase of DRESS.^22^

The aging of the world population is associated with a greater frequency of comorbidities and concomitant use of different drugs. Added to that there is an increasing number of new molecules constantly added to the pharmacopeia. These elements warn for a likely increase in the incidence of DRESS and the importance of its early recognition and treatment.

## Financial support

None declared.

## Authors' contributions

Hélio Amante Miot: Design and planning of the study, drafting and editing of the manuscript and approval of the final version of the manuscript.

Roberta Fachini Jardim Criado: Design and planning of the study, drafting and editing of the manuscript and approval of the final version of the manuscript.

Carolina Talhari: Design and planning of the study, drafting and editing of the manuscript and approval of the final version of the manuscript.

Paulo Ricardo Criado: Design and planning of the study, drafting and editing of the manuscript and approval of the final version of the manuscript.

Paulo Müller Ramos: Design and planning of the study, drafting and editing of the manuscript and approval of the final version of the manuscript.

Mayra Ianhez: Design and planning of the study, drafting and editing of the manuscript and approval of the final version of the manuscript.

## Conflicts of interest

Paulo Ricardo Criado: Advisory board - Pfizer, Galderma, Takeda, Hypera, Novartis, Sanofi; Clinical research - Pfizer, Novartis, Sanofi, Amgen and Lilly; Speaker: Pfizer, Abbvie, Sanofi-Genzyme, Hypera, Takeda, Novartis.

Roberta Fachini Jardim Criado: Advisory board - Pfizer, Takeda, Hypera, Novartis, Sanofi; Clinical Research - Pfizer, Novartis, Sanofi and Lilly; Speaker: Pfizer, Abbvie, Sanofi-Genzyme, Hypera, Takeda, Novartis.

Hélio Amante Miot: Advisory Board – Johnson & Johnson, L’Oréal, Theraskin, Sanofi and Pfizer; Clinical Research - Abbvie, Galderma and Merz.

Mayra Ianhez: Advisory Board- Galderma, Sanofi, Pfizer, Novartis, Abbvie, Janssen; Speaker - Galderma, Sanofi, Pfizer, Theraskin, Novartis, Abbvie, Janssen, Leopharma, FQM.

Paulo Müller Ramos: Speaker and Advisory Board – Pfizer.

Carolina Talhari: No conflicts of interest.
